# Sulfate Resistance of Recycled Aggregate Concrete with GGBS and Fly Ash-Based Geopolymer

**DOI:** 10.3390/ma12081247

**Published:** 2019-04-16

**Authors:** Jianhe Xie, Jianbai Zhao, Junjie Wang, Chonghao Wang, Peiyan Huang, Chi Fang

**Affiliations:** 1 School of Civil and Transportation Engineering, Guangdong University of Technology, Guangzhou 510006, China; jhxie@gdut.edu.cn (J.X.); gdutzjb@hotmail.com (J.Z.); gdutwch@hotmail.com (C.W.); fangchi1993@hotmail.com (C.F.); 2 Division of Engineering, NYU Abu Dhabi, P.O. Box 129188, Abu Dhabi, UAE; 3 School of Civil Engineering and Transportation, South China University of Technology, Guangzhou 510640, China; pyhuang@scut.edu.cn

**Keywords:** recycled aggregates, geopolymer concrete, sulfate attack, hydration mechanism, ground granulated blast furnace slag, fly ash

## Abstract

There is a constant drive for the development of ultra-high-performance concrete using modern green engineering technologies. These concretes have to exhibit enhanced durability and incorporate energy-saving and environment-friendly functions. The object of this work was to develop a green concrete with an improved sulfate resistance. In this new type of concrete, recycled aggregates from construction and demolition (C&D) waste were used as coarse aggregates, and granulated blast furnace slag (GGBS) and fly ash-based geopolymer were used to totally replace the cement in concrete. This study focused on the sulfate resistance of this geopolymer recycled aggregate concrete (GRAC). A series of measurements including compression, X-ray diffraction (XRD), and scanning electron microscopy (SEM) tests were conducted to investigate the physical properties and hydration mechanisms of the GRAC after different exposure cycles in a sulfate environment. The results indicate that the GRAC with a higher content of GGBS had a lower mass loss and a higher residual compressive strength after the sulfate exposure. The proposed GRACs, showing an excellent sulfate resistance, can be used in construction projects in sulfate environments and hence can reduce the need for cement as well as the disposal of C&D wastes.

## 1. Introduction

The new global standards of modern engineering technologies, continuously requiring more energy-saving and environment-friendly infrastructure [1,2], are driving the development of recycled construction materials. Traditional concrete, as one of the largest construction materials worldwide, entails a significant consumption of both energy and raw materials and, consequently, causes serious environmental pollution [3]. Therefore, it is imperative to minimize these negative impacts on the environment. Recently, increasing attention has been paid to researches on the use of supplementary cementitious materials [4,5,6] and construction and demolition (C&D) waste [7,8,9,10]. 

As an environmentally friendly material, geopolymer, has been found to be an ideal substitute for ordinary Portland cement (OPC) [11,12,13]. Geopolymer, first described by Davidovits [14], is an inorganic polymer material with a three-dimensional network structure, which can be prepared by the alkali-activated polymerization of silica tetrahedron and alumina tetrahedron from industrial waste [15]. Fly ash [12,16,17], GGBS [18,19], calcined clays [20,21], and other by-products [22,23] from recycled waste, as geopolymers, have been investigated by different researchers. It is well accepted in these studies that geopolymer concrete is able to provide a better mechanical performance, durability, and environmental protection than ordinary concrete.

In addition, transforming C&D waste into aggregates in concrete production is also considered a promising recycling technology, promoting the reuse of solid waste and consequently reducing not only environmental pollution, but also the consumption of raw materials [24,25]. This may be one of the significant efforts required for achieving sustainable construction. However, because recycled aggregates are characterized by a high porosity, high water absorption, and low strength, concrete containing a high content of recycled aggregates exhibits an inferior performance, compared to ordinary concrete [26,27]. Therefore, extensive efforts have been devoted to increasing the performance of recycled aggregate concretes (RC) [9,28,29]. Among the reported approaches, using geopolymer to replace OPC is believed to be one of the most promising engineering technologies to improve the mechanical properties of RC [9]. This is because, in addition to the defects of recycled aggregates themselves, most of the damage caused to concrete can be traced back to chemical and mechanical defects in the cement structure [30], which can be modified by the outstanding chemical and physical properties of geopolymer. Previous studies [9,17,31,32,33,34] focused on the mechanical or microstructural characteristics of GRAC. It was found that the combination of geopolymer and recycled aggregate concrete (GRAC) not only provides a significant enhancement of the internal matrix of RC, but also reduces environmental impact, absolutely promoting the development of recycled construction materials. However, it should be mentioned that very limited information is available on the durability aspects of GRAC, especially concerning its sulfate resistance.

In fact, sulfate attack is one of the main durability problems for concrete structures, especially concrete in environments with sulfate ions [35]. The ingress of sulfate ions contributes to the formation of ettringite and cracks when a critical stress, generated by the expansion force of ettringite, is reached. Thus, sulfate attack could cause spalling, cracking, and decreased strength of concrete structures. The sulfate resistance was also reported to be worse in RC, compared to ordinary concrete (NC), when a high content of recycled aggregates was used [36,37,38,39]. Therefore, how to prevent or reduce the damage of sulfate attack to concrete structures and improve the sulfate corrosion resistance of recycled concrete has aroused widespread concern [40,41]. A few studies have shown that geopolymer can provide a better sulfate resistance to ordinary concrete than OPC [42,43]. Fernandez–Jimenez et al. [42] soaked the fly ash-based geopolymer in Na_2_SO_4_ solution for one year and found that the compressive strength of the specimens increased continuously. Hardjito et al. [43] also reported similar experimental results. It is noted that no substantial work has been performed on the sulfate resistance of alkali-activated GRAC.

## 2. Research Significance

The demand for reducing the detrimental impact of C&D waste on the environment and addressing the shortage of natural resources places a burden on the government. Additionally, as the main binder in conventional concrete, Portland cement has been reported as the second most consumed product on Earth, second only to water, and its production is expected to increase in the coming years. To reduce the need for cement as well as the disposal of C&D wastes, using C&D waste and geopolymer together, as a green and eligible alternative to conventional concrete, is a promising technology. Numerous studies have reported on the performance of geopolymer concrete. However, the majority of these studies focused on natural aggregate concrete, with a limited number on recycled concrete. Additionally, these previous studies focused on the independent influence of fly ash on the mechanical properties of geopolymer concrete [44], with limited information on the coupling effects of GGBS and fly ash on the performance of geopolymer concrete, especially recycled concrete. In fact, some researchers have found that single fly ash-based geopolymer concrete may exhibit a low early strength, which has been recognized as an apparent disadvantage [45,46]. In order to improve the mechanical properties of concrete with single fly ash-based geopolymer, the incorporation of GGBS into it was reported to be an effective method [47,48]. Therefore, it is necessary to develop a green concrete that incorporates recycled aggregates and GGBS and fly ash-based geopolymer. 

In order to reduce the consumption of natural resources [49,50,51], recycle the waste materials [52,53,54,55,56], and effectively apply this new type of concrete in infrastructure exposed to soils, groundwater, rivers, seawater, and industrial wastes containing high concentrations of sulfate ions, it is essential to study its sulfate resistance. To the best of the authors’ knowledge, no data are available in the literature on the sulfate resistance of recycled aggregate concrete with GGBS and fly ash-based geopolymer. The object of this work is to develop this environment-friendly GRAC, with an improved sulfate resistance. In this study, recycled aggregates from construction and demolition (C&D) waste are used as coarse aggregates, and GGBS and fly ash-based geopolymer is used to totally replace cement in concrete. A series of specimens were prepared by considering different amounts of GGBS and sulfate exposure durations. They were then exposed to a sulfate environment and tested under uniaxial compression. The coupling effects of the GGBS and fly ash-based geopolymer and the recycled coarse aggregates are examined in terms of the sulfate resistance of GRAC. The mass loss, crack propagation, and residual compressive strength are analyzed. The optimum content of GGBS in GRAC, based on the experimental results, is also discussed. Finally, XRD and SEM tests are conducted to reveal the attack mechanisms of the sulfate ions and hydration mechanisms of GRAC.

## 3. Experiment Set-Up

### 3.1. Materials

**Figure 1:**
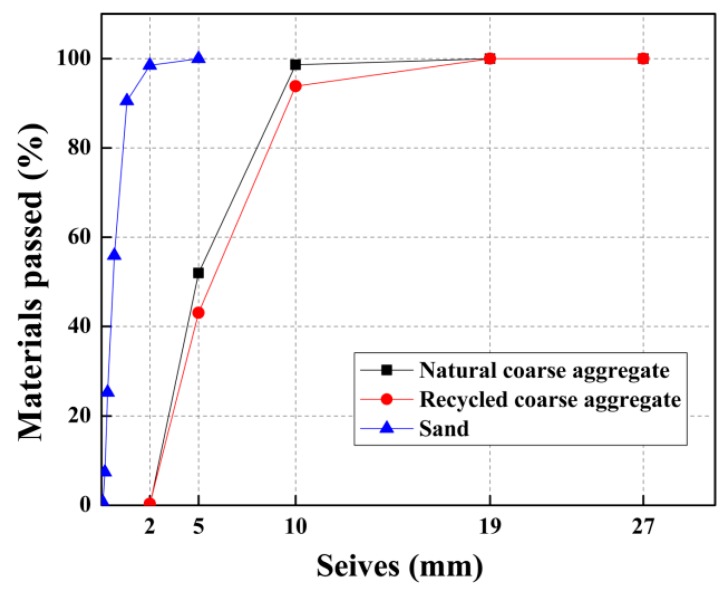
Grading curves of the aggregates used.

In this study, recycled coarse aggregates, produced by crushed waste concrete, were used. The recycled coarse aggregates were obtained from demolished buildings. The original concrete was characterized by a compressive strength of 20–30 MPa. To improve the purity and quality of recycled coarse aggregates, a high-pressure (5 MPa) water jet was used to remove the mud in recycled aggregates. Wood and glass impurities were removed manually. Then, they were dried by sunshine before use. The ingredient proportions in the recycled coarse aggregates are presented in Table 1, which met the requirements of class III of recycled aggregates, according to the Chinese standard GB/T 25177-2010 [57]. For comparison, natural coarse aggregates, made of granite, were used in the control specimen (ordinary concrete). Both natural coarse aggregates and recycled coarse aggregates have a continuous grain size distribution of between 5 and 20 mm. The main properties of coarse aggregates are summarized in Table 2. River sand was extracted as fine aggregates, with a fineness modulus of 2.52, a water absorption rate of 0.8%, and a specific gravity of 2.69. The size grading of all aggregates is presented in Figure 1.

Ordinary Portland cement (OPC), with a strength of 42.5 MPa, complying with the Chinese standard GB175-2007 [58], was used in the control specimen. The properties and chemical compositions of the OPC, fly ash, and GGBS were shown in Table 3.

The alkaline activator used in this study was a mixture of NaOH and Na_2_SiO_3_ solutions. The properties of NaOH and Na_2_SiO_3_ solutions are listed in Table 4 and Table 5, respectively. The solid NaOH used for NaOH solution had a purity of 99%. Following the findings in the previous literature [43,59,60,61], the mass ratio of NaOH to Na_2_SiO_3_ solution was 1:2.5. 

To improve the setting time of GRAC, a retarding water reducer (RWR), consisting mainly of calcium sucrose, was used in this study. The properties of the RWR were reported in Table 6.

In total, 20 groups of test specimens were considered in this study. There were 5 mixes (see Table 7), which were exposed to 4 different cycles of sulfate attack (see Table 8). Each group included 3 replicas of test specimens, with dimensions of 100 mm × 100 mm × 100 mm. In all mixes, the water-binder ratio (W/B) was kept constant and equal to 0.5. The specimens NC and RC in Table 7 were OPC-based concrete with natural aggregates and recycled aggregates, respectively. In geopolymer concretes, 3 levels of GGBS content were considered. The RWR solution in all mixes was fixed at 1.5%, according to the flowability. The slump test results show that the combination of GGBS and fly ash can provide acceptable workability for the geopolymer concrete with recycled coarse aggregates. The consistency of GRAC is significantly influenced by GGBS content. An increase of GGBS content can reduce the slump value of GRAC. A detailed discussion of this can be found in our previous study [48]. In terms of the curing condition, the geopolymer specimens, S25, S50, and S75, were demolded after 24 h and cured under steam and a high temperature of 80 °C for another 24 h, before being put under a standard curing condition (20 °C and RH > 95%). The specimens, NC and RC, were cured under the standard curing condition, until testing.

### 3.2. Test Procedures

The test of sulfate attack in this study was conducted according to the Chinese standard GBT 50082 [62]. At the age of 26 days, the specimens were oven-dried (80 ± 5 °C) for 24 h, and then, after they were cooled down to room temperature, the weight of each specimen was measured (*m_1_*). Subsequently, the specimens were put into a wetting–drying chamber, containing sulfate solution with 5% Na_2_SO_4_. During the wetting stage, the level of the solution was at least 20 mm higher than the top surface of the specimens. Each wetting stage lasted for 15 h. During the drying stage, the specimens were air-dried for 30 min and then dried at 80 °C for 6 h. They were then cooled down to room temperature for 2 h. Each wetting–drying cycle lasted for around 24 h. The sulfate solution was replaced weekly in order to maintain the same sulfate content and pH level. This is because the migration of alkali from the concretes to the solution could increase the pH of the sulfate solution noticeably [63]. After the targeted cycles of attack, the specimens were oven-dried (80 ± 5 °C) for 24 h and cooled down to room temperature. The weight was then measured (*m*_2_), and the compressive strength was tested. 

To investigate the residual compressive behavior, after exposure to sulfate attack, all specimens were tested under axial compression loading at room temperature. The compression test was conducted by the Italian MATEST material testing machine (Matest, Treviolo, Bergamo, Italy), with a capacity of 4000 kN. The axial load, controlled by the stress, was applied at a speed of 0.5 MPa/s.

To examine the hydration mechanism and sulfate resistance of GRAC, X-ray diffraction (XRD), and Scanning Electron Microscopy (SEM) tests were conducted for the samples, from different depths (0–5 mm, 20–25 mm, and 45–50 mm) below the surface of specimens. Copper was used as the anode material. For each measurement, 1.5 g of the powder sample, passed through a sieve with an aperture of 48 µm, was used for XRD measurement with a Bruker D8 ADVANCE X-Ray Powder Diffractometer (Bruker, Karlsruhe, Baden-Wü rttemberg, Germay). The scanning rate was 4°/min and from 4° to 80° (2θ) for a continuous measurement. 

As for SEM observations, an electron microscopy, LYRA 3 XMU (Tescan, Brno, Southern Moravia, Czech Republic), made by TESCAN was used in this study. The magnification could be adjusted from 1 to 1000 thousand times. The SEM samples were prepared by extraction from the crack surface of specimens, with a size of less than 10 mm × 10 mm × 10 mm. They were carefully classified and stored in sealed plastic bags, before SEM observation. Additionally, the spray gold coating was implemented to make the concrete samples electronically conductive, which enabled their microstructures and micro scale damage to be observed in SEM.

## 4. Results and Discussions

### 4.1. Mass Loss after Exposure to Sulfate Attack

To quantify the mass loss, the ratio *K_m_* between the mass of the specimen, before and after the sulfate attack, is used.

*K_m_* = *m*_2_/*m*_1_ × 100 (1)

where *m*_1_ and *m*_2_ are the weight of the specimen, before and after the sulfate attack, respectively.

**Figure 2:**
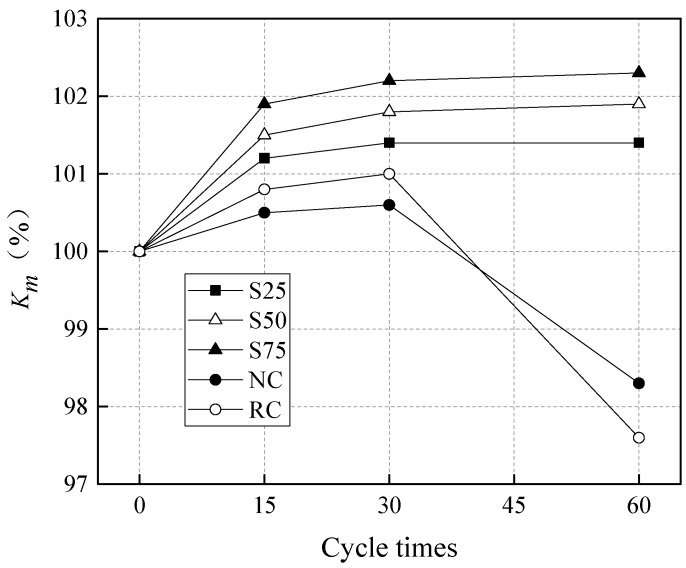
The influence of sulfate attack cycles on the mass loss of specimens.

Figure 2 presents the effects of sulfate attack cycles on the mass loss of GRAC. It can be seen from Figure 2 that before sulfate attack, all geopolymer concretes showed less weight, compared to the NC and RC. It is an advantage to have less self-weight for geopolymer concretes, because the self-weight could take a significant part of the load, and this needs to be considered by engineers. As for NC and RC, the weight of RC was less than NC. This is because the recycled coarse aggregates in RC had less weight than the natural coarse aggregates in NC due to the existence of the porous cement paste in the recycled aggregates. 

As demonstrated in Figure 2, the *K_m_* values of geopolymer concretes, S25, S50, and S75, were all higher than 100, and the weight of these specimens increased gradually, with the increase of sulfate attack cycles, up to 60 cycles. As for RC and NC, the weight of specimens only increased continuously up to 30 cycles of sulfate attack, and from 30 to 60 cycles, the weight decreased significantly. The weight increase in specimens is attributed to the formation of new minerals due to the sulfate attack. The mineral characterization will be discussed later through XRD analysis. The mass loss in RC and NC, after 30 cycles of sulfate attack, is caused by the broken and fallen pieces from the surface of specimens due to sulfate attack. These findings indicate that GRAC is characterized by a better resistance to sulfate attack than both RC and NC.

Figure 2 shows that the *K_m_* values of geopolymer concretes increased initially and became stable with the increase of sulfate attack cycles. From 0 to 15 cycles, 15 to 30 cycles, and 30 to 60 cycles, the *K_m_* values of the S25 group increased by 1.2%, 0.2%, and 0.0%, the *K_m_* values of the S50 group increased by 1.5%, 0.3%, and 0.1%, and the *K_m_* values of the S75 group increased by 1.9%, 0.3%, and 0.1%, respectively. It can be seen that the *K_m_* values of GRAC increased with the increase of the GGBS content, especially from 0 to 15 cycles of sulfate attack. This observation could be explained by the fact that the Ca^2+^ content in GGBS is greater than that in fly ash, and the SO_4_^2−^ in the sulfate solution could react with the Ca^2+^ and the aluminum phases to form CaSO_4_ and ettringite, increasing the density of the geopolymer concrete. For NC and RC, the *K_m_* values of RC after 15 and 30 cycles were higher than those of NC, but the *K_m_* value of RC after 60 cycles was lower than that of NC, as demonstrated in Figure 2. The initial higher increase of weight in RC may be due to the RC having more pores to protect the newly formed minerals from sulfate attack. The latter higher mass loss after 30 cycles in RC is due to the microstructure in RC being weaker than NC and forming cracks more easily. In addition, there was more expandable minerals formed in RC, leading to the broken pieces in RC being more likely to fall off. Besides, the other reason is that the C-S-H phases decalcify in the cement paste with the increase of sulfate exposure, as reported in a literature [64].

### 4.2. Appearance Change after Exposure

**Figure 3:**
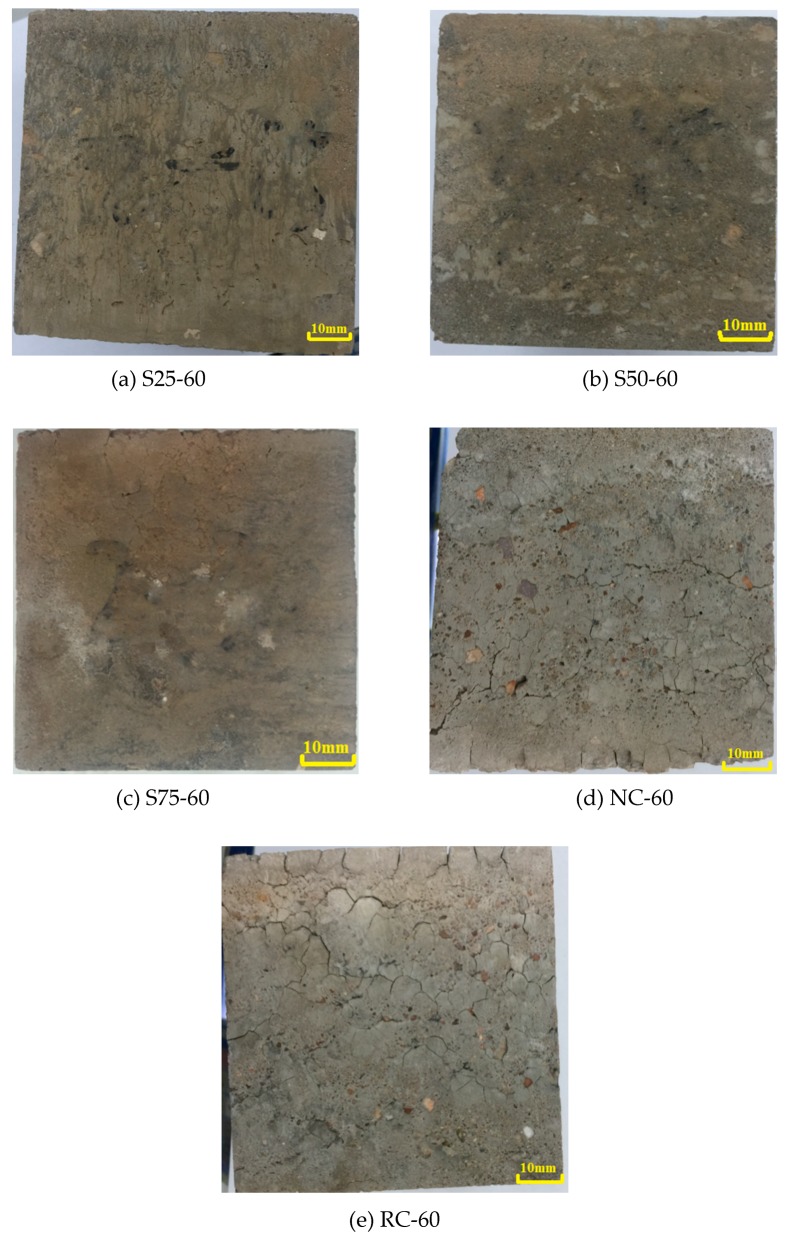
Appearance of specimens after 60 cycles of sulfate attack.

The specimen appearances are studied after experiencing sulfate attack. Figure 3 shows the distribution of surface cracks in typical specimens after exposure to 60 cycles of sulfate attack. As observed, the GRAC had less cracks on the surface, compared to RC and NC. There were some fine cracks on the surface of the S25 group geopolymer concrete, and there were less cracks in the S50 and S75 groups. In RC and NC, there were a lot of cracks, and the crack width was large. The RC group had more cracks than the NC. 

In NC, the cracks propagated in a line-like pattern. The cracks in NC were supposed to start from the interfacial transition zone (ITZ) between the cement matrix and the aggregates and then propagate along the direction of the initial cracks. In RC, the cracks propagated in a random map pattern, and it seems that the cracks were formed randomly and propagated along a closed shape. This finding suggests that there were more defects in terms of crack propagation in RC than in NC, when they were under sulfate attack. Figure 3 also shows that, in the GRACs, it was hard to see any large cracks like those in RC and NC. These observations indicate that the RC had a worse resistance to sulfate attack than NC. However, when the cement paste was replaced with geopolymer, the recycled concrete could be modified by GGBS and fly ash-based geopolymer, resulting in a better resistance to sulfate attack than NC.

### 4.3. Residual Compressive Strength after Sulfate Attack

The ratio *K_f_* between the compressive strength, before and after sulfate attack, is usually used to describe the residual compressive strength.

*K_f_* = *f_cn_*/*f*_*c*0_ × 100 (2)

where *f*_*c*0_ and *f_cn_* are the compressive strength of the specimen, before and after sulfate attack, respectively.

**Figure 4:**
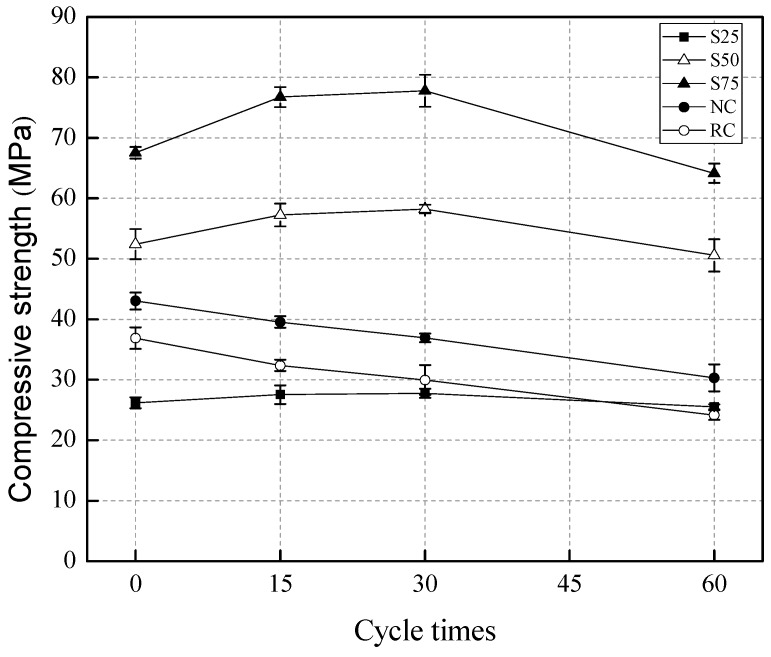
The influence of sulfate attack cycles on the compressive strength of specimens.

**Figure 5:**
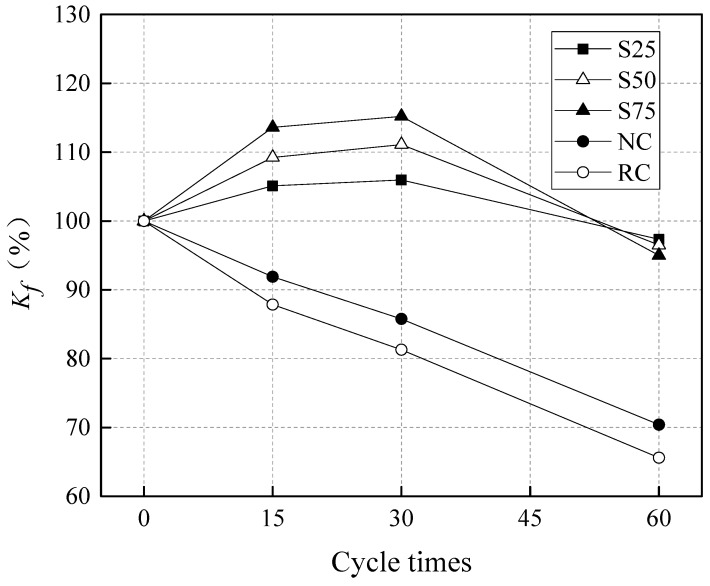
The influence of sulfate attack cycles on the *K_f_* of specimens.

The influence of sulfate attack cycles on the compressive strength and *K_f_* was shown in Figure 4 and Figure 5. It can be seen that the compressive strength of geopolymer concretes, S25, S50, and S75, increased after 15 and 30 cycles of sulfate attack, but decreased after 60 cycles of sulfate attack. For NC and RC, the compressive strength decreased continuously from 15 to 60 cycles of sulfate attack. These findings show that geopolymer concretes have a stronger resistance to sulfate attack than NC and RC. The initial increase of concrete compressive strength in the GRAC after 15 and 30 cycles of sulfate attack can be attributed to the following causes.

1. The GRACs had less porosity and a denser microstructure, compared to NC and RC. The ingress of sulfate ions was slower in geopolymer concretes than in NC and RC.
2. The initial ingress of sulfate ions contributed to the filling of the micro-pores by the formation of CaSO_4_ and ettringite. This can enhance the microstructure of the GRACs and thus increase their compressive strength.
3. The GRACs had less porosity and a denser microstructure, compared to NC and RC. The ingress of sulfate ions was slower in geopolymer concretes than in NC and RC. The high-temperature drying at 80 °C for 6 h in the drying stage at every cycle of sulfate attack could accelerate the hydration process of the initially unreacted GGBS and fly ash in geopolymer concretes. Besides, the free water trapped in the micro-pores can be evaporated at high temperatures, leaving space for additional hydration products.

From 30 to 60 cycles, Figure 5 demonstrates that the compressive strength of the GRACs decreased significantly, which can be attributed to the microstructure being destroyed due to the increased cycles of sulfate attack. At this stage, the high-temperature drying treatment might make the destroyed microstructure worse. As for the RC and NC groups, their compressive strength decreased continuously from 0 to 60 cycles of sulfate attack. These finding indicate that the porosity of RC and NC should be more than that of the geopolymer concretes, and the microstructure of RC and NC was weaker, compared to the geopolymer concretes. Interestingly, it can be found, from Figure 4, that S75 had an increase of 165% and 112% in the residual compressive strength after 60 cycles of sulfate attack, compared to RC and NC, respectively. This can also be attributed to the cross-linked aluminosilicate polymer matrix being more stable than the OPC paste in NC and RC [65,66]. Besides, the sodium hydroxide contributed to the enhancement of the strength and durability of the geopolymer concrete [17]. Similar results for natural aggregate concrete with a geopolymer binder were reported in the literature [67,68,69].

For geopolymer concretes with different GGBS contents, Figure 5 shows that, after 15 and 30 cycles of sulfate attack, the *K_f_* of S25 increased by 5.1% and 11.1%, the *K_f_* of S50 increased by 6.0% and 13.6%, and the *K_f_* of S75 increased by 9.2% and 15.2%, respectively. This result indicates that the compressive strength of the geopolymer recycled concretes with a higher GGBS content was higher after the initial sulfate attack. This phenomenon can be explained by the fact that there was more *Ca^2+^* in the GGBS than in the fly ash, and there were more reactions between the GGBS and the sulfate solution. However, it is noted that, after 60 cycles, the *K_f_* of S25, S50, and S75 decreased by 2.7%, 3.5%, and 5.0% respectively. This finding indicates that, although there was a higher increasing rate of compressive strength in S75 mixes at the initial stage of sulfate attack (before 15 cycles), the decreasing rate of compressive strength of S75 mixes at the later stage of sulfate attack (after 30 cycles) was also higher than that of other mixes. For NC and RC, the *K_f_* of RC was always lower than that of NC after 15, 30, and 60 cycles of sulfate attack. That is to say, the sulfate resistance of RC is worse than that of NC. The reason for this observation is that there are more defects in RC, compared to NC. 

### 4.4. Mineral Characterization (XRD Results)

**Figure 6:**
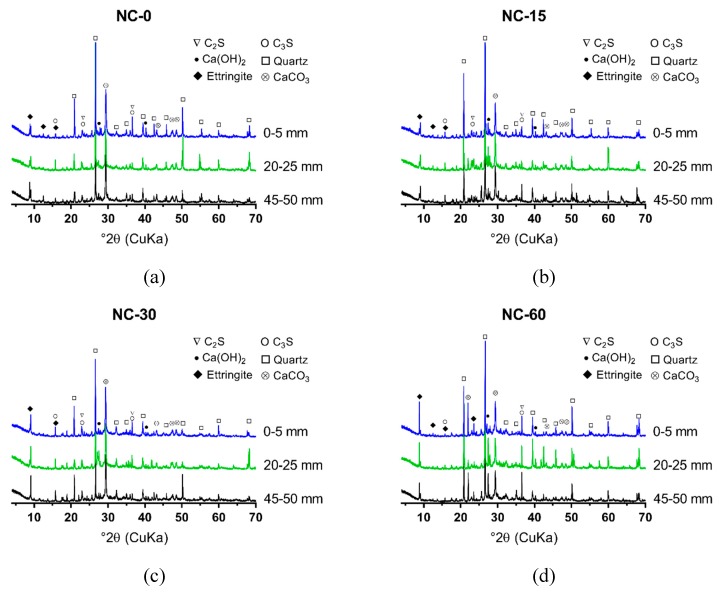
X-ray diffraction (XRD) spectrum of the powder samples from ordinary concrete (NC), after 0, 15, 30, and 60 cycles of sulfate attack (sample location distance to the attack surface: 0–5 mm, 20–25 mm, and 45–50 mm): (**a**) NC-0; (**b**) NC-15; (**c**) NC-30; (**d**) NC-60.

**Figure 7:**
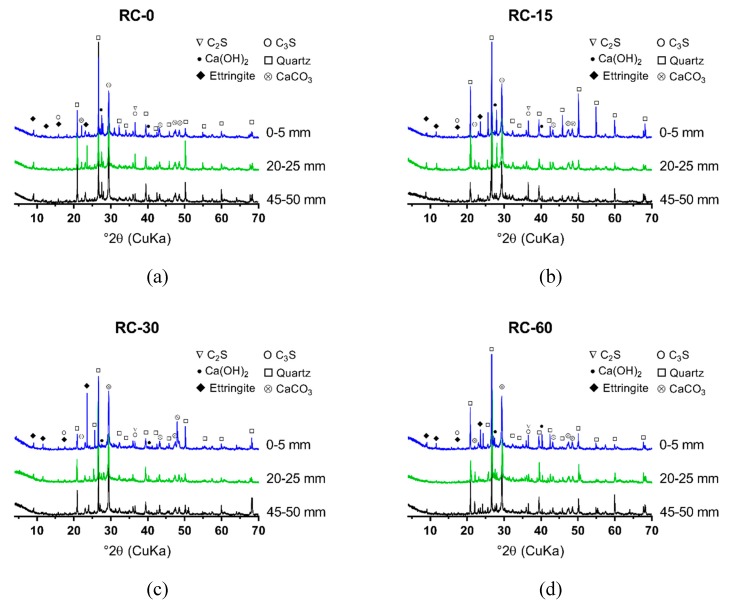
XRD spectrum of the powder samples from aggregate concretes (RC), after 0, 15, 30, and 60 cycles of sulfate attack (sample location distance to the attack surface: 0–5 mm, 20–25 mm, and 45–50 mm): (**a**) RC-0; (**b**) RC-15; (**c**) RC-30; (**d**) RC-60.

**Figure 8:**
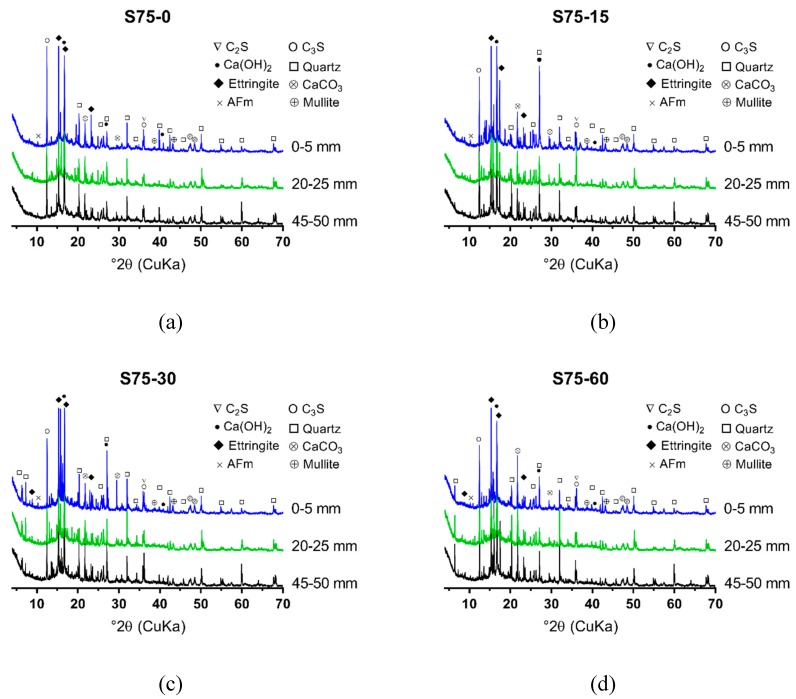
XRD spectrum of the powder samples from S75, after 0, 15, 30, and 60 cycles of sulfate attack (sample location distance to the attack surface: 0–5 mm, 20–25 mm, and 45–50 mm): (**a**) S75-0; (**b**) S75-15; (**c**) S75-30; (**d**) S75-60.

The XRD results of NC, RC and S75, after 0, 15, 30, and 60 cycles of sulfate attack, are shown in Figure 6, Figure 7 and Figure 8. The powders were collected at 3 depths from the attack surface to investigate the change of minerals at different depths due to sulfate attack. A comparison of Figure 6, Figure 7 and Figure 8 shows that, from 0 to 30 cycles of sulfate attack, there is no significant difference in the amount of ettringite at different depths; after 30 cycles, the amount of ettringite is higher at a lower depth from the attack surface. This finding suggests that the formation of additional ettringite on the surface or the inside, close to the surface, due to the ingress of sulfate ions, contributed to the cracks that formed, as shown in Figure 3. 

In NC and RC, the ettringite on the attack surface did not change much from 0 to 15 cycles of sulfate attack, but it increased significantly after 30 cycles of sulfate attack. The formation of CaCO_3_ also increased with the increase of the cycles of sulfate attack. The initial sulfate attack might contribute to a denser surface of the specimens because of the ettringite that formed and filled the pores inside. This process was slow, because the initial increase rate of ettringite was low before 15 cycles of sulfate attack. Thus, the aforementioned decrease of strength in NC and RC after 15 cycles of sulfate attack is likely not to have been caused by the ettringite, but may be attributed to the effect of high temperature (80 °C) during the wetting–drying cycles. After 15 cycles of sulfate attack, the formation of ettringite was accelerated, and cracks formed (Figure 3) in the specimens, especially on the surface. With the increase and further propagation of cracks, more sulfate ions penetrated into a deeper position from the surface of the specimen, and more ettringite was formed, which caused further expansion and more cracks.

With the increase of the GGBS content, a large quantity of ettringite initially formed in GRAC, before the exposure of sulfate attack, which was different from NC and RC. The ettringite that initially formed in S75 was due to the reaction between the aluminum phase and sulfate phase in activated GGBS and fly ash. The ettringite and aluminum gel that formed in GRAC contributed to it having a denser microstructure, compared to NC and RC. This contributed to a decreased in the ingress rate of sulfate ions into the geopolymer concrete samples. From 0 to 60 cycles of sulfate attack, visible cracks on the surface of S75 specimens did not increase significantly, which indicated that the dense microstructure in S75 concretes can defer the formation and accumulation of additional ettringite, formed due to sulfate attack. 

### 4.5. SEM Results

**Figure 9:**
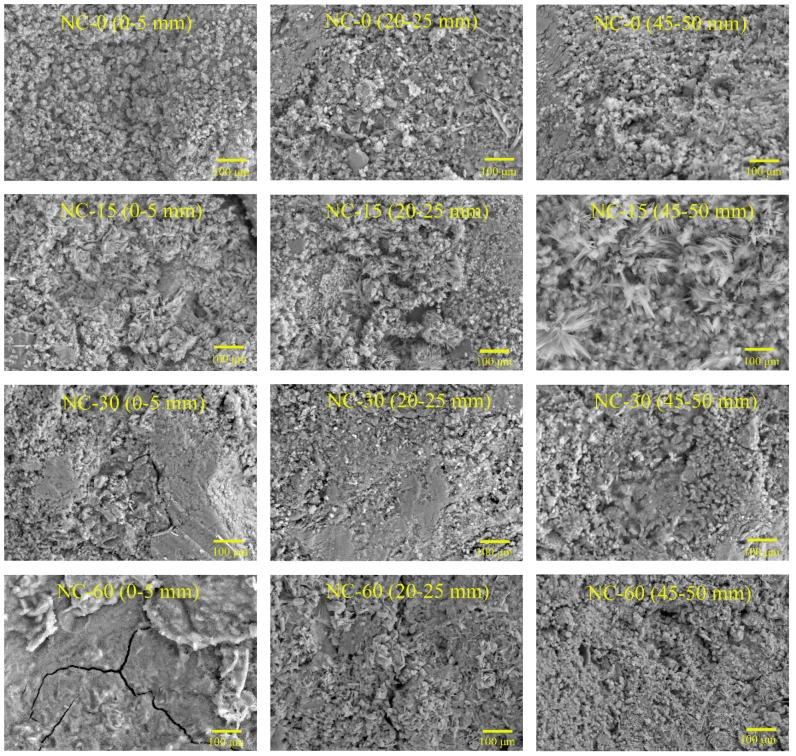
Scanning electron microscopy (SEM) images of NC concretes, after 0, 15, 30, and 60 cycles of sulfate attack (sample location from attack surface: 0–5 mm, 20–25 mm, and 45–50 mm).

**Figure 10:**
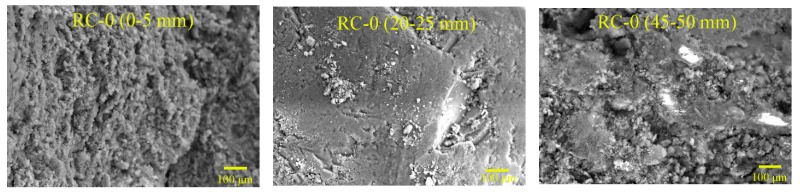

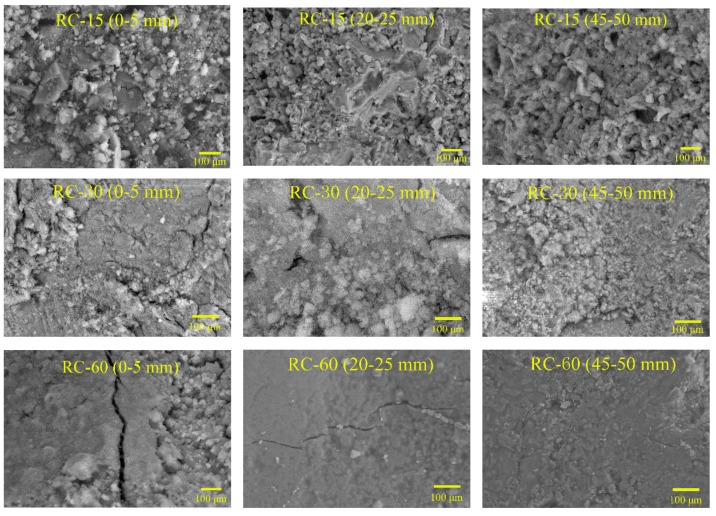
SEM images of RC concretes, after 0, 15, 30, and 60 cycles of sulfate attack (sample location from attack surface: 0–5 mm, 20–25 mm, and 45–50 mm).

**Figure 11:**
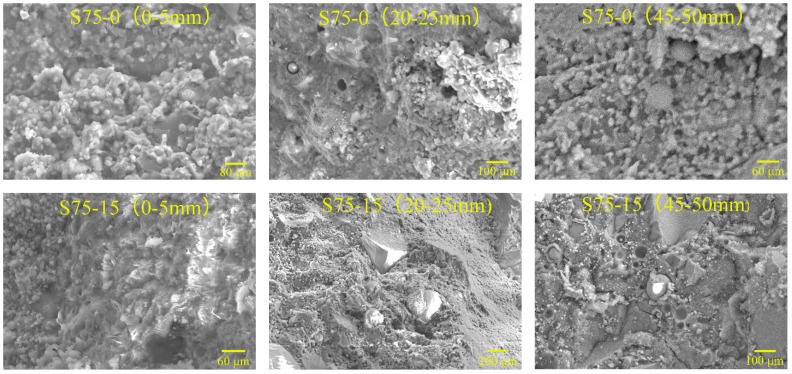

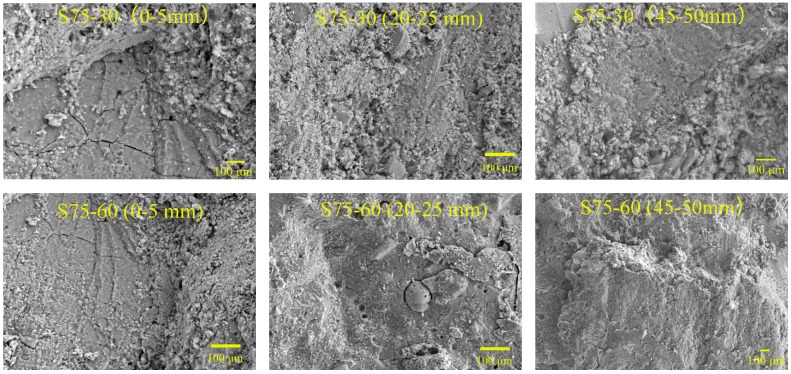
SEM images of S75 concretes, after 0, 15, 30, and 60 cycles of sulfate attack (sample location from attack surface: 0–5 mm, 20–25 mm, and 45–50 mm).

The microstructures of NC, RC, and S75, after different cycles of sulfate attack, are shown in Figure 9, Figure 10 and Figure 11. The SEM samples were prepared by extraction from the sulfate attack surface of the specimen at a depth of 0–5 mm, 20–25 mm, and 45–50 mm. It can be seen, from Figure 9, Figure 10 and Figure 11, that before sulfate attack the micro-structure of S75 was much denser than that of NC and RC. The cracks in RC appeared earlier than those in NC and S75 geopolymer concretes. As for NC and RC, the micro-cracks formed after 15 cycles of sulfate attack, and the crack width increased rapidly with the increase of sulfate attack cycles.

As shown in Figure 11, no cracks in the S75 specimen were caused by the sulfate attack on the surface at 0–5 mm, after 15 cycles of sulfate attack. After 30 cycles of sulfate attack, micro-cracks formed at 0–5 mm and 20–25 mm in S75 concretes. In comparison with the compressive strength results, shown in Figure 4, it is suggested that these micro-cracks did not result in an obvious decrease of strength. After 60 cycles of sulfate attack, the crack width increased and propagated significantly, compared to that at 30 cycles of sulfate attack. These cracks resulted in a decrease of compressive strength of S75 concretes, as shown in Figure 4.

A comparison of Figure 9 and Figure 10 shows that more cracks formed in RC, compared to NC, after 30 cycles of sulfate attack. This can be attributed to RC having many defects, such as the cracks in recycled aggregates and the old ITZ between old cement paste and recycled aggregates, as well as the new ITZ between OPC and recycled aggregates. However, the GRAC specimen, S75, showed micro-cracks only after 30 cycles of sulfate attack. Even after 60 cycles of sulfate attack, S75 still showed less and smaller cracks, compared with NC and RC. There are two possible reasons for this difference. First, the geopolymer matrix contributed to the enhancement of the ITZ properties between the matrix and recycled aggregates. The other reason is that the GGBS and fly ash-based geopolymer matrix has a better resistance to sulfate attack than the OPC matrix in NC and RC.

## 5. Conclusions

To develop a green concrete with an improved sulfate resistance, recycled aggregate concrete, with GGBS and fly ash-based geopolymer, was prepared in this study. An experimental study was conducted to investigate the sulfate resistance of this GRAC. A series of compression, XRD, and SEM tests were conducted to investigate the physical properties and hydration mechanisms of the GRAC, after different sulfate attack durations. The following conclusions can be drawn from the experimental results:

1. GGBS and fly ash-based geopolymer can provide an excellent sulfate resistance for recycled concrete. Compared with cement-based concrete, GRAC exhibits a lower mass loss, lower crack propagation, and a higher residual compressive strength. Therefore, GRAC is ideally suitable to be used in construction projects in sulfate environments and hence reduces the need for cement, as well as the disposal of C&D wastes.
2. GGBS and fly ash have a good coupling effect in the improvement of the sulfate resistance of GRAC. The specimen with GGBS and fly ash was relatively intact after exposure to sulfate attack. The SEM analysis also shows that GGBS and fly ash geopolymer can improve the compactness of recycled concrete, thereby enhancing the bonding property between the paste and aggregates.
3. The sulfate resistance of GRAC increases with the increase of the GGBS content. Compared to cement-based concrete, the GRAC incorporating GGBS and fly ash, with a proportion of 3:1, might have an increase by more than double in residual compressive strength after 60 cycles of sulfate attack.
4. The initiation of the internal cracks in the cement-based recycled concrete commonly occurred in the old ITZ and the new ITZ, i.e., between the old aggregates and old cement matrix in recycled aggregates and between the new cement matrix and recycled aggregates, respectively. As for the GRAC, the ITZ between the recycled aggregates and the new matrix was enhanced with the increase of the GGBS content, and the geopolymer matrix could prevent crack propagation, unlike the OPC matrix.
5. Unlike the cement-based concrete, the residual compressive strength of the GRAC with a high GGBS content did not show a monotonous decrease with the increase of the sulfate attack duration. Interestingly, the residual strength of the GRAC showed a trend of first increasing and then decreasing with the increase of exposure time. The SEM and XRD analysis indicate that this initial improvement is attributed to the formation of additional ettringite, which filled the pores inside the concrete and consequently improved the compactness of the concrete, while too much ettringite caused crack initiation, resulting in the reduction of compressive strength.

## Figures and Tables

**Table 1:** The proportions of components in recycled aggregates.

Granite Aggregates	Red Bricks	Old OPC Pastes	Broken Tiles
78.9%	10.4%	8.4%	2.1%

**Table 2:** The properties of natural coarse aggregates and recycled coarse aggregates.

Aggregates	Particle Size (mm)	Apparent Density (kg/m^3^)	Bulk Density (kg/m^3^)	Water Absorption (%)	Crushing Index (%)
Natural aggregates	5–20	2677	1318	0.9	9.7
Recycled aggregates	5–20	2270	1400	6.0	11.0

**Table 3:** The properties and chemical compositions of granulated blast furnace slag (GGBS), fly ash, and ordinary Portland cement (OPC). (Reproduced from the previous work [46] with permission from Elsevier).

Indexes	GGBS	Fly Ash	OPC
**Specific surface area (m^2^/kg)**	400	600	-
**Density (g/cm^3^)**	2.8	2.34	-
**Fineness (80 µm)**	-	-	1.1
**SiO_2_ (%)**	35.52	51.49	20–24
**Al_2_O_3_ (%)**	13.60	24.36	4–7
**CaO (%)**	35.05	9.8	62–67
**Fe_2_O_3_ (%)**	0.61	5.49	5–6
**SO_3_ (%)**	1.72	2.14	2.12
**MgO (%)**	9.58	1.2	0.9
**Ignition loss (%)**	3	2.34	1.7

**Table 4:** Properties of NaOH solution. (Reproduced from the previous work [46] with permission from Elsevier).

Concentration (mol/L)	Density (g/cm^3^)	NaOH (wt%)	H_2_O (wt%)
8	1.275	25.1	74.9

**Table 5:** Properties of Na_2_SiO_3_ solution. (Reproduced from the previous work [46] with permission from Elsevier).

Modulus	Degrees Baumé (°Bé)	Density (g/cm^3^)	Na_2_O (wt%)	SiO_2_ (wt%)	H_2_O (wt%)
3.34	39~41	1.387	7.3	27.6	65.0

**Table 6:** Properties of the retarding water reducer. (Reproduced from the previous work [46] with permission from Elsevier).

PH (1% Weight Solution)	Water Reducing (wt%)	Air Content (vol%)	Solid Content (wt%)	Insoluble Content (wt%)	Setting Time (min)
12 ± 1	8	<5.5%	≥92	≤5%	>+90

**Table 7:** Mix proportions. (Reproduced from the previous work [46] with permission from Elsevier).

Mix	Mix Proportions (kg/m^3^)												
	S%	W/B	NCA	RCA	SA	OPC	S	F	W	NaOH	Na_2_SiO_3_	AW	RWR
NC	-	0.5	1225	-	525	400	-	-	200	-	-	-	6
RC	-	0.5	-	1225	525	400	-	-	200	-	-	74	6
S25	25%	0.5	-	1225	525	-	100	300	200	78	195	74	6
S50	50%	0.5	-	1225	525	-	200	200	200	78	195	74	6
S75	75%	0.5	-	1225	525	-	300	100	200	78	195	74	6

Notes: S%, percentage of GGBS by weight; W/B, water-binder ratio; NCA, natural coarse aggregates; RCA, recycled coarse aggregates; SA, river sand; OPC, ordinary Portland cement; S, GGBS; F, flay ash; W, total water, including water in the retarding water-reducer, NaOH solution and Na_2_SiO_3_ solution; NaOH, prepared NaOH solution; Na_2_SiO_3_, prepared Na_2_SiO_3_ solution; AW, additional water needed for the pre-wetting of RCA, according to the water absorption of RCA, shown in Table 2; RWR, retarding water-reducer.

**Table 8:** Specimen group and test conditions.

Mix	Specimen Group	Cycle Times
NC	NC-0	0
	NC-15	15
	NC-30	30
	NC-60	60
RC	RC-0	0
	RC-15	15
	RC-30	30
	RC-60	60
S25	S25-0	0
	S25-15	15
	S25-30	30
	S25-60	60
S50	S50-0	0
	S50-15	15
	S50-30	30
	S50-60	60
S75	S75-0	0
	S75-15	15
	S75-30	30
	S75-60	60

## Abbreviations

C&D	construction and demolition
GGBS	granulated blast furnace slag
GRAC	geopolymer recycled aggregate concrete
NC	ordinary concrete
OPC	ordinary Portland cement
RC	recycled aggregate concrete
SEM	Scanning Electron Microscopy
XRD	X-ray diffraction
